# Raised D-dimer among Admitted COVID-19 Patients in a Tertiary Care Centre: A Descriptive Cross-sectional Study

**DOI:** 10.31729/jnma.7579

**Published:** 2022-07-31

**Authors:** Dipesh Karki, Roshani Gurung, Prasanna Nepali, Hari Prasad Kaphle, Bhabuk Subedi, Sundar Adhikari

**Affiliations:** 1 Department of Medicine, Fishtail Hospital and Research Center Private Limited, Pokhara, Kaski, Nepal; 2 Pharmacy Programme, Gandaki University, Pokhara, Kaski, Nepal; 3 Fishtail Hospital and Research Center Private Limited, Pokhara, Kaski, Nepal; 4 School of Health and Allied Sciences, Pokhara University, Pokhara, Kaski, Nepal; 5 Department of Intensive Care Unit, Fishtail Hospital and Research Center Private Limited, Pokhara, Kaski, Nepal; 6 Department of Pharmacy, Fishtail Hospital and Research Center Private Limited, Pokhara, Kaski, Nepal

**Keywords:** *COVID-19*, *D-dimer*, *Nepal*, *prevalence*

## Abstract

**Introduction:**

Patients with COVID-19 are characterised by abnormal levels of inflammatory biomarkers. Elevated D-dimer in COVID-19 patients is associated with increased mortality. This study aimed to find out the prevalence of raised D-dimer among COVID-19 patients in a tertiary care centre.

**Methods:**

This descriptive cross-sectional study was conducted in COVID-19 unit of a tertiary care centre from 23 January 2021 to 19 June 2021. The ethical approval was taken from the Institutional Review Committee (Reference number: 077/078/159). D-dimer values and demographic data of the hospital-admitted COVID-19 patients were recorded. Convenience sampling technique was used. Point estimate and 95% Confidence Interval were calculated.

**Results:**

Out of 180 patients with COVID-19 admitted in the hospital, the D-dimer levels were raised in 85 (47.22%) (39.93-54.51, 95% Confidence Interval) patients.

**Conclusions:**

The prevalence of raised D-dimer among admitted COVID-19 patients was found to be lower when compared to other studies conducted in similar settings.

## INTRODUCTION

Patients with Coronavirus Disease-2019 (COVID-19) are characterised by abnormal levels of inflammatory biomarkers such as C-reactive protein (CRP), ferritin, fibrinogen and especially elevated level of D-dimer which indicates need for critical care and may lead to death.^1^

D-dimer is a fibrin degradation product (fibrinolysis) that circulates in blood plasma at low blood concentration where value less than 0.5 μg/ml is usually considered normal.^2^ D-dimer value is used for the investigation of venous thromboembolism, pulmonary embolism and now list has been added to the investigation of COVID-19 patients because the level of D-dimer rises with increased severity of community acquired pneumonia.^3^ It was first reported by physician in Wuhan, China, found to be independently associated with mortality having D-dimer >1 μg/ml.^4,5^

This study aimed to find out the prevalence of raised D-dimers among COVID-19 patients in a tertiary care centre.

## METHODS

This descriptive cross-sectional study was conducted in COVID-19 unit of a tertiary care centre from 23 January 2021 to 19 June 2021 at the peak time of COVID-19 second wave in Nepal. The Institutional Review Committee of Fishtail Hospital and Research Centre Private Limited, Pokhara, Nepal, provided the ethical approval for the study (Reference number: 077/078/159). Hospitalised patients with confirmed COVID-19 by reverse transcription polymerase chain reaction (RT-PCR) using nasopharyngeal swab were included in this study. Patients with the diagnosis of cancer, hematologic malignancy, acute coronary syndrome, presence of other infections, chronic liver disease, trauma or surgery within 30 days, patients with prior use of anticoagulants, pregnancy, patients without prior recorded D-dimer upon admission and cases without recorded outcomes were excluded from the study. Convenience sampling was done, and sample size was calculated by using the following formula:


$$\begin{array}{ll} \text{n} & ={\text{Z}}^{2}\times \frac{\text{p}\times \text{q}}{{\text{e}}^{\text{2}}}\\ & =1.96^{2}\times \frac{0.50\times 0.50}{0.08^{2}}\\ & =150 \end{array}$$

Where,

n = minimum required sample size
Z = 1.96 at 95% of Confidence Interval (CI)
p = prevalence taken as 50% for maximum sample size calculation
q = 1-p
e = margin of error, 8%

The calculated sample size was 150. Taking a 20% non-response rate, the sample size was 180.

The reference level for raised D-dimer level is ≥0.5 μg/ml.^5^ The demographic data of the respondents were taken as gender and age. Age was categorised into three classes as the age group from 20 to 39, 40 to 59, and 60 or above. For the assessment of D-dimer, blood samples were collected within 24 hours of admission and sent to the laboratory department of the hospital. Analysis was done on I chroma (manufactured in South Korea) by the Fluorescence Immunoassay (FIA) method. All measurements were done in the laboratories within 2 hours of sample collection.

For data entry and analysis, Microsoft Excel and IBM SPSS Statistics 20.0 was used. Point estimate and 95% CI were calculated.

## RESULTS

Out of 180 patients with COVID-19 admitted in the hospital, the D-dimer levels were raised in 85 (47.22%) (39.93-54.51, 95% CI) patients. Among the patients with elevated D-dimer, 31 (36.47%) patients expired (Table 1).

**Table 1:** Treatment outcome in COVID-19 patients with elevated D-dimer (n = 85).

Treatment outcome	n (%)
Discharge on patient request/Discharged	54 (63.53)
Expired	31 (36.47)

Majority of them were of age group 40-59 years 37 (43.53%) followed by 60 and above 35 (41.18%) respectively (Table 2).

**Table 2:** Age distribution in COVID-19 patients with elevated D-dimer (n = 85).

Age (years)	n (%)
20-39	13 (15.29)
40-59	37 (43.53)
60 and above	35 (41.18)

**Figure 1:**
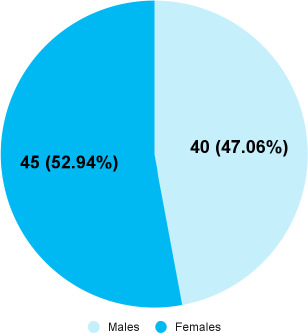
Sex distribution in COVID-19 patients with elevated D-dimer (n= 85).

Among the admitted patients with COVID-19, the raised D-dimer levels were found in 45 (52.94%) female patients (Figure 1).

## DISCUSSION

In this study, the prevalence of raised D-dimer was found to be 47.22%. Among them 36.47% of the patients died. The ongoing pandemic of COVID-19 poses several challenges to clinicians. To control this situation, many allopathic medicines have been used in practice but no conclusive treatment has arrived. On another side, it is found to have an advantageous effect of different medicinal plants which are traditionally used as antiviral, and anti-inflammatory properties and possesses phytochemicals like alkaloids, glycosides, phenol, flavonoid etc. on COVID-19 treatment.^7-10^

Due to rapid disease progression, causing severe and fatal complications, it is necessary to screen COVID-19 patients for biomarkers, categorization of patients, their clinical management, and prevention of serious complications. Biomarkers commonly evaluated to access severity of COVID-19 diseases are D-dimer, serum ferritin, C-reactive protein (CRP), Interleukin-6, Lactate dehydrogenase (LDH), and CT Severity score derived from a patient's CT imaging.^6,11^

D-dimers are one of the fragments produced when plasmin cleaves fibrin to break down clot. It is the smallest protein fragment identified in blood when a blood clot is degraded (fibrinolysis). In previous studies, it is found that higher the D-dimer level, the patient is more severe with higher mortality rate which might be associated with the severity of inflammation prior coagulopathy/thrombosis that lead to poor outcomes, even death.^12,13^ Similar case has also been seen in our study, the mortality rate was higher with the patients having D-dimer value >0.5 μg/ml. Most of the patients with mortality were female (52.94%) where mortality rate for male was (47.06%) and age group 40-59 years (43.53%) followed by 60 and above (41.18%). This study was supported by the previous studies.^4-5^ A published study reported that COVID-19 patient admitted to the intensive care units has elevated level of D-dimer value.^14^ Also, study conducted in 279 COVID-19 patients in three hospitals in Hubei Province, China shows the strong relationship between D-dimer level and the progression of COVID-19.^15^

This was a single-centred study. In the future, it is necessary to do such a study with a larger sample in multiple centres. Association between the parameters could not be established. Furthermore, other biomarkers can also be compared for the severity assessment and outcome of a patient's treatment with COVID-19.

## CONCLUSIONS

The prevalence of raised D-dimer among admitted COVID-19 patients was found to be lower when compared to other studies conducted in similar settings. In this study, the mortality rate of COVID-19 patients with raised D-dimer was over one third. However, it is necessary to do further research to clarify risk of severity in COVID-19 patients with elevated D-dimers which are the result of thrombotic burden or tendency, inflammation, or a combination therein.
